# Attractor cortical neurodynamics, schizophrenia, and depression

**DOI:** 10.1038/s41398-021-01333-7

**Published:** 2021-04-12

**Authors:** Edmund T. Rolls

**Affiliations:** 1 Oxford Centre for Computational Neuroscience, Oxford, UK; 2 Department of Computer Science, University of Warwick, Coventry, UK

**Keywords:** Schizophrenia, Depression

## Abstract

The local recurrent collateral connections between cortical neurons provide a basis for attractor neural networks for memory, attention, decision-making, and thereby for many aspects of human behavior. In schizophrenia, a reduction of the firing rates of cortical neurons, caused for example by reduced NMDA receptor function or reduced spines on neurons, can lead to instability of the high firing rate attractor states that normally implement short-term memory and attention in the prefrontal cortex, contributing to the cognitive symptoms. Reduced NMDA receptor function in the orbitofrontal cortex by reducing firing rates may produce negative symptoms, by reducing reward, motivation, and emotion. Reduced functional connectivity between some brain regions increases the temporal variability of the functional connectivity, contributing to the reduced stability and more loosely associative thoughts. Further, the forward projections have decreased functional connectivity relative to the back projections in schizophrenia, and this may reduce the effects of external bottom-up inputs from the world relative to internal top-down thought processes. Reduced cortical inhibition, caused by a reduction of GABA neurotransmission, can lead to instability of the spontaneous firing states of cortical networks, leading to a noise-induced jump to a high firing rate attractor state even in the absence of external inputs, contributing to the positive symptoms of schizophrenia. In depression, the lateral orbitofrontal cortex non-reward attractor network system is over-connected and has increased sensitivity to non-reward, providing a new approach to understanding depression. This is complemented by under-sensitivity and under-connectedness of the medial orbitofrontal cortex reward system in depression.

## Introduction

A computational neuroscience approach to the stability of attractor networks in the cortex is described, and then it is shown how differences in the operation of these systems are related to schizophrenia and depression. Attractor networks are neuronal networks prototypical of the neocortex and hippocampus that have associatively modifiable recurrent collateral synaptic connections between the pyramidal cells. Such networks are the way in which the brain implements long-term memory, short-term memory, the source of the top-down bias for attention, and decision-making^1–4^.

The computational neuroscience approach taken here involves modeling cortical systems at the level of integrate-and-fire neurons with synaptically activated ion channels in attractor or autoassociation networks implemented with the recurrent collateral connections^1,3,4^. This enables us to link from effects expressed at synapses and ion channels, through their effects on the spiking neuronal activity in the network and the noise that this introduces into the system, to global effects of the network such as the stability of short-term memory, attentional, and decision-making systems, and thus to cognitive function, dysfunction, and behavior. This provides a unifying approach to many aspects of cortical function, which helps in the understanding of short-term memory, long-term memory, top-down attention, decision-making, executive function, and the relation between the emotional and the reasoning systems in the brain^1,3–6^. This approach in turn leads to new approaches based on the stability of neurodynamical systems to some psychiatric disorders including schizophrenia and depression^4,7–13^, and to how changes in glutamate and GABA function may contribute to the symptoms and mechanisms of these disorders. This approach in turn leads to suggestions for treatments.

I first introduce this computational neuroscience approach, and then consider how it can be applied to schizophrenia and depression.

## Attractor networks, and their stability

The attractor framework is based on dynamical systems theory. In a network of interconnected neurons, a memory pattern (represented by a set of active neurons) can be stored by synaptic modification, and later recalled by external inputs. Furthermore, a population of neurons firing in the network activated by an input is then stably maintained by the system even after input offset. The population of neurons firing could represent memories, perceptual representations, or thoughts, depending on the cortical region involved^1,3,4^.

**Fig. 1:**
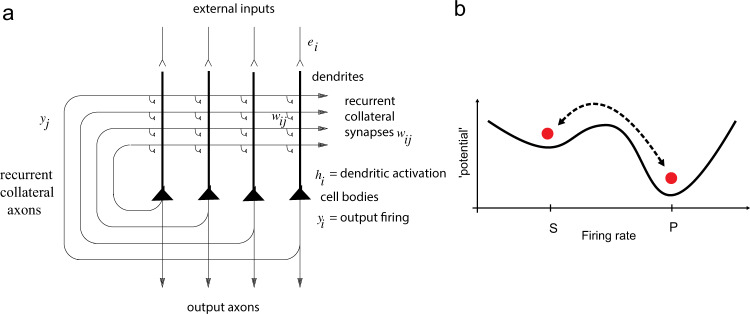
Attractor Networks. **a** Architecture of an attractor network. External inputs *e*_*i*_ activate the neurons in the network, and produce firing *y*_*i*_, where *i* refers to the *i*’th neuron. The neurons are connected by recurrent collateral synapses *w*_*ij*_, where *j* refers to the *j*th synapse on a neuron. By these synapses, an input pattern on *e*_*i*_ is associated with itself, and thus the network is referred to as an autoassociation network. Because there is positive feedback via the recurrent collateral connections, the network can sustain persistent firing. These synaptic connections are assumed to be formed by an associative (Hebbian) learning mechanism. The inhibitory interneurons are not shown. They receive inputs from the pyramidal cells and make negative feedback connections onto the pyramidal cells to control their activity. The recall state (which could be used to implement short-term memory or memory recall) in an attractor network can be thought of as the local minimum in an energy landscape. **b** Energy landscape. The first basin (from the left) in the energy landscape is the spontaneous state with a low firing rate (S), and the second basin is the high firing rate attractor state, which is ‘persistent’ (P) in that the neurons that implement it continue firing at a high rate. The vertical axis of the landscape is the energy potential. The horizontal axis is the firing rate, with high to the right. In the normal condition, the valleys for both the spontaneous and for the high firing attractor state are equally deep, making both states stable. In general, there will be many different high firing rate attractor basins, each corresponding to a different memory. In schizophrenia, it is hypothesized that the high firing rate (P) state is too shallow due to low firing rates, providing instability which leads to the cognitive symptoms of poor short-term memory and attention in the prefrontal cortex. It is also hypothesized that in schizophrenia the spontaneous firing rate state (S) is too shallow due to reduced inhibition and that this leads to noise-induced jumping into high-firing rate states in the temporal lobe that relate to the positive symptoms of schizophrenia such as hallucinations and delusions. In contrast, it is hypothesized that in obsessive–compulsive disorder, the basin for the high firing attractor state is deep, making the high firing rate attractor state that implements for example short-term memory too stable, and very resistant to distraction^29^. This increased depth of the basin of attraction of the persistent state may be associated with higher firing rates of the neurons if for example the state is produced by increased currents in NMDA receptors^29^.

**Fig. 2:**
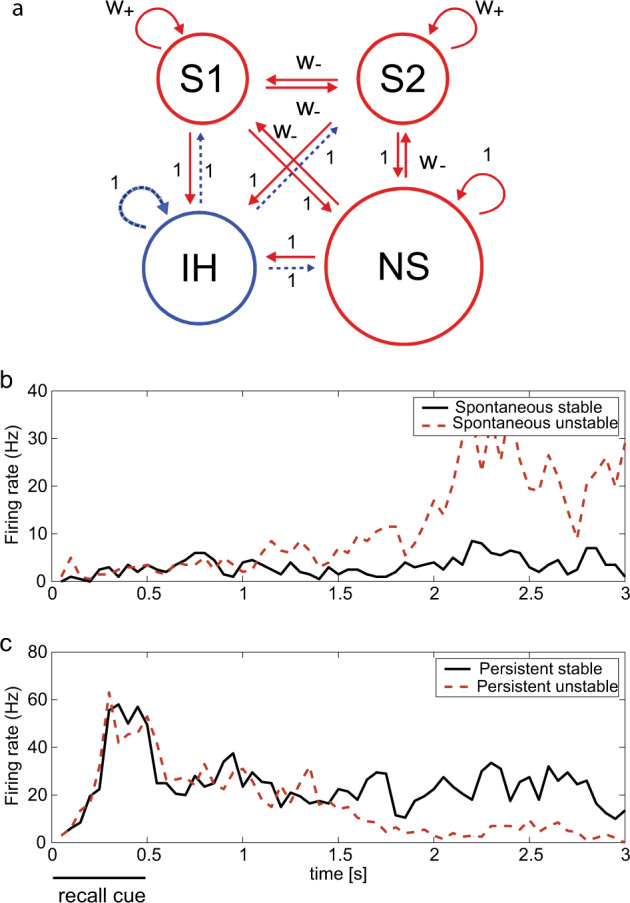
Stability and Instability in Attractor Networks. **a** The attractor network model. The excitatory neurons (red) are divided into two selective pools S1 and S2 (with 40 neurons each) with strong intra-pool connection strengths *w*_+_ and one non-selective pool (NS) (with 320 neurons). The other connection strengths are 1 or weak *w*_−_. The network contains 500 neurons, of which 400 are in the excitatory pools and 100 are in the inhibitory pool IH (blue). Each neuron in the network also receives inputs from 800 external neurons, and these neurons increase their firing rates to apply a stimulus or distractor to one of the pools S1 or S2. The synaptic connection matrices are provided elsewhere^4,7,29^. **b** Example trials of the integrate-and-fire attractor network simulations of short-term memory. The average firing rate of all the neurons in the S1 neuronal population (or pool) is shown. Performance without a recall cue. The spontaneous firing rate is maintained at a low rate correctly on most trials (spontaneous stable), but on some trial the spiking-related noise in the network triggers the S1 population of neurons into a high firing rate state (spontaneous unstable), which is incorrect. **c** Performance with a recall cue applied to S1 at 0–500 ms. In the stable persistent (i.e., short-term memory) type of trial, the firing continues or persists at a moderate rate throughout the trial after the end of the recall cue (persistent stable), and this is correct. On some trials, the spiking-related noise provokes a transition to the low firing rate state, and this is incorrect (persistent unstable). In these simulations, the network parameter was *w*_+_ = 2.1. (Modified from Loh et al.^7^).

**Fig. 3:**
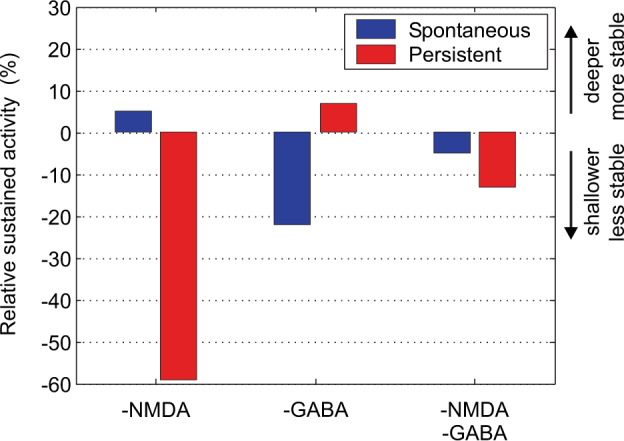
Stability of the spontaneous low firing rate and persistent high firing rate states of the short-term memory in the integrate-and-fire attractor network of Fig. 2. The percentage of trials in which the average activity during the last second (2–3 s) remained in the reference state is shown. Decreasing the NMDA conductances by 4.5% (NMDA: −1) decreased the stability of the high-firing rate (Persistent) state, in that the firing often failed to be maintained in the high firing rate short-term memory state. Decreasing the GABA conductances by 9% decreased the stability of the Spontaneous firing rate state, with the system frequently jumping into a high firing rate state. Decreasing both the NMDA and the GABA conductances decreased the stability of the high firing rate short-term memory state (labeled persistent), which frequently fell out into low firing. Decreasing both the NMDA and the GABA conductances in addition decreased the stability of the Spontaneous state, which sometimes jumped into a high firing rate state. The condition of decreased NMDA and GABA is how we characterize schizophrenia, in that stability of attention, memory, and thought processes implemented by high firing rate states are reduced as applies to the cognitive symptoms; and in that the system often jumps from the spontaneous low firing rate state in which there is no retrieval cue into a high firing rate state, modeling the positive symptoms with intrusive thoughts, delusions, and hallucinations. Shallower basins of attraction relate to less stability. (Modified from Loh et al.^7^).

**Fig. 4:**
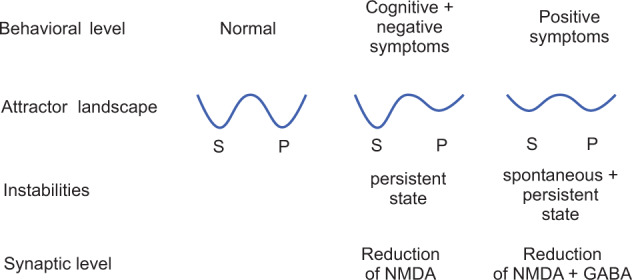
Summary of the attractor hypothesis of schizophrenic symptoms and simulation results (see text). The first basin (from the left) in each energy landscape is the low firing rate spontaneous state (S), and the second basin is the persistent (or continuing) high firing rate attractor state (P). The horizontal axis of each landscape is the firing rate, increasing to the right. The vertical axis of each landscape is the energy potential. (Modified from Loh et al.^7^).

The architecture of an attractor or autoassociation network is as follows (see Fig. 1a). External inputs *e*_*i*_ activate the neurons in the network, and produce firing *y*_*i*_, where *i* refers to the *i*’th neuron. The neurons are connected to each other by recurrent collateral synapses *w*_*ij*_, where *j* refers to the *j*’th synapse on a neuron. By these synapses, an input pattern on *e*_*i*_ is associated with itself, and thus the network is referred to as an autoassociation network. Because there is positive feedback implemented via the recurrent collateral connections, the network can sustain persistent firing. These synaptic connections are assumed to build up by an associative (Hebbian) learning mechanism^14^. The inhibitory interneurons are not shown. They receive inputs from the pyramidal cells and make inhibitory negative feedback connections onto the pyramidal cells to keep their activity under control. Hopfield^15^ showed that the recall state in a simple attractor network can be thought of as the local minimum in an energy landscape, where the energy would be defined as

1 $$E = - \frac{1}{2}\mathop {\sum}\limits_{i,j} {w_{ij}\left( {y_i - < y > } \right)\left( {y_j - < y > } \right)}$$

where <..> indicates the ensemble average. The concept is that a particular attractor implemented by a subset of the neurons in a network will have low energy, and be stable if the neurons *i* and *j* within the attractor are connected by strong synaptic weights *w*_*ij*_ and have high firing rates *y*_*i*_ and *y*_*j*_. Autoassociation attractor systems have two types of stable fixed points: a spontaneous state with a low firing rate, and one or many more attractor states with high firing rates in which the positive feedback implemented by the recurrent collateral connections maintains a high firing rate (Fig. 1b). We sometimes refer to this latter state as the persistent state (see P in Figs. 1–4). The area in the energy landscape within which the system will move to a stable attractor state is called its basin of attraction. The number of different attractor states, each represented by a subpopulation of the neurons firing, is in the order of the number of recurrent collateral synaptic connections (C) onto each neuron, if sparse distributed representations are used^4,16^. Given that C is in the order of 10,000 in the neocortex and hippocampus, approximately 10,000 different memories could be stored in a single attractor network, extending across approximately 2 mm in the neocortex^4^.

The attractor dynamics can be pictured by energy landscapes, which indicate the basin of attraction by valleys, and the attractor states or fixed points by the bottom of the valleys. (Although energy functions apply to recurrent networks with symmetric connections between the neurons^15^ as would be the case in a fully connected network with associative synaptic modification, and do not necessarily apply to more complicated networks with for example incomplete connectivity, nevertheless the properties of these other recurrent networks are similar^3,4,7,16–19^, and the concept of an energy function and landscape is useful for discussion purposes.)

The stability of an attractor is characterized by the average time in which the system stays in the basin of attraction under the influence of noise. The noise provokes transitions to other attractor states. One source of noise results from the random (Poissonian) spiking times of individual neurons for a given mean rate and the finite-size effect due to the limited number of neurons in the network^1,3,4,20^, and another source might be distracting stimuli.

To investigate whether noise is still present with the larger networks present in the brain, a new series of studies has been performed. First, the noise tends to decrease as the size of networks, the number of neurons in the network, is increased. We simulated large integrate-and-fire attractor networks with several thousands of neurons and showed that finite-size effects still apply, that is, that noise still significantly influences the operation of the system^19–21^. Second, neurons in the cortex typically have graded firing rates, with each neuron having an approximately exponential distribution of firing rates to a set of stimuli^3,4,22,23^. We simulated large integrate-and-fire attractor networks with graded firing rate representations and found that the noise was greater than for the networks with binary (high or low) firing rates normally studied^21^. Third, the connectivity between neurons in the cortex is typically diluted, with the probability of connections between any pair of even nearby pyramidal cells in the range of 0.1–0.04^3,4,19^. We simulated large integrate-and-fire attractor networks with diluted connectivity and showed that dilution, achieved by having more neurons in the network but maintaining constant the number of recurrent collateral connections onto each neuron, decreased the noise in the network^19^. Overall, these investigations showed that biologically plausibly large integrate-and-fire networks with graded firing rate representations and diluted connectivity typical of the cortex still show effects of the spiking noise from individual neurons on their performance. These investigations are thus important in showing that noise is an important factor in influencing biologically plausible cortical attractor networks^4,19,21^.

To illustrate what can be revealed by this type of analysis we simulated an integrate-and-fire attractor network with spiking neurons with approximately Poisson spike times so that there was noise in the system (Fig. 2a)^4,7,8^. The network simulations investigated the stability of short-term memory against noise in the system using a recall cue on ‘Persistent’ trials at time 0–0.5 s in a 3 s trial. Examples of the operation of the system are shown in Fig. 2b, c. The spontaneous state (S) in which no memory recall cue is applied should remain in a low firing rate state, but sometimes due to spiking-related noise in the system jumps incorrectly into a high firing rate state. When a recall cue is applied in the persistent state (P), the system should remain stable in a high firing rate state of persistent activity, but sometimes, incorrectly, fails to maintain the short-term memory and falls into a low level of firing. Figure 3 shows that decreasing the (excitatory) NMDA receptor activations decreased the stability of the high firing rate attractor (P, Persistent) state (and decreased the firing rates), in that the high firing rate state persisted on fewer trials. Decreasing GABA, which is inhibitory on the excitatory system and increased the firing rates, made the spontaneous state (S) less stable, in that it tended to stay in the spontaneous state for a shorter time, and jumped incorrectly into the high firing rate state. With NMDA and GABA both reduced, the stability of the high firing rate state P was reduced in that sometimes the network dropped out of the high firing short-term memory state; and also the stability of the spontaneous (S) state in which no memory recall cue had been applied was reduced, in that the network sometimes jumped into a high firing rate state. The third condition models aspects of schizophrenia, as described below. The details of these simulations are described elsewhere^4,7,8^.

## Schizophrenia

### A top-down computational neuroscience approach to schizophrenia

We have adopted a *top-down* approach that considers whether generic alterations in the operation and stability of cortical circuits in different cortical areas might lead to the different symptoms of schizophrenia^1,2,4,7–10,24^. Bottom-up approaches start with putative changes at the neural level such as alterations in dopamine and try to understand the implications for function^25,26^. The top-down approach complements the bottom-up approaches, as it starts from the set of symptoms and maps them onto a dynamical systems computational framework. The approach described here is to produce a neurally based mechanistic model that can elucidate the phenomena experienced by patients.

**Fig. 5:**
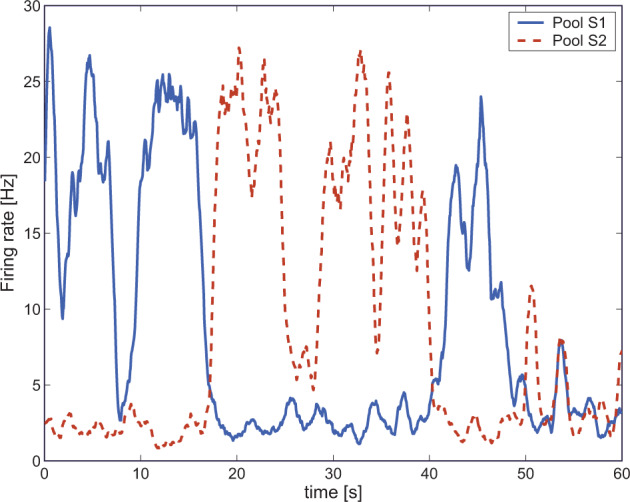
Wandering between attractor states by virtue of statistical fluctuations caused by the randomness of the spiking activity. We simulated a single long trial (60 s) in the spontaneous test condition for reduced NMDA and reduced GABA synaptic efficacy (NMDA: −1, GABA: −1). The two curves show the activity of the two selective pools S1 and S2 over time smoothed with a 1 s sliding averaging window. The activity moves noisily between the attractor for the spontaneous state and the two high firing rate persistent attractor states S1 and S2. (Modified from Loh et al.^7^).

The stochastic dynamical systems approach that we utilize includes integrate-and-fire neurons with currents passing through voltage-dependent and hence non-linear ion channels activated by NMDA receptors, and currents through ion channels activated by AMPA and GABA receptors^1,3,4,7–9,27^. The positive feedback in the recurrent collateral connections in the network, the NMDA receptor non-linearity, and the non-linearity introduced by the threshold for the firing of the neurons in the system, provide the system with non-linearities that enable it to have the properties of an attractor network (see Figs. 2–5)^1,4,6,7,9,20,28,29^.

### Neurodynamical hypotheses of schizophrenia

The neurodynamical hypotheses of schizophrenia described next relate to and are supported by evidence that in schizophrenia there may be reduced (excitatory) cortical glutamate transmission^30^, which may relate in part to reduced dendritic spine density^30–32^. Drugs that act at the glycine-modulatory site of the NMDA receptor to increase cortical glutamatergic transmission may be useful in the treatment of some symptoms of schizophrenia^33^. Consistent evidence is that administration of treatments such as ketamine that block NMDA receptors can be associated with acute positive and negative-like symptoms of schizophrenia^34^. In addition, disturbances of GABA-mediated inhibition may be present in schizophrenia^31,35^ and may lead to overactivity of some excitatory neurons, as described below. Excitatory D1 receptors in the prefrontal cortex are implicated in working memory by facilitating memory delay-related neuronal firing, and their activation is a potential treatment for cognitive symptoms in schizophrenia^36^.

#### Cognitive symptoms

The cognitive symptoms of schizophrenia include distractibility, poor attention, and the dysexecutive syndrome^37–40^. The core of the cognitive symptoms of schizophrenia is a working-memory deficit characterized by a difficulty in maintaining items in short-term memory implemented in the dorsolateral prefrontal cortex^31,41–43^. The impairments of attention induced by prefrontal cortex damage may be accounted for in large part by an impairment in the ability to hold the object of attention stably and without distraction in the short-term memory systems in the prefrontal cortex^4,44,45^.

Specific simulations of impairments in the operation of prefrontal attractor networks can help to explain how the cognitive symptoms of schizophrenia, including poor short-term memory, poor ability to allocate and maintain attention, and distractibility, occur. We have proposed that the working-memory and attentional deficits might be related to instabilities of the high-firing states in attractor networks in the prefrontal cortex (Fig. 4)^7,8,29^. Specifically, NMDA receptor hypofunction, which has been associated with schizophrenia^30,46–50^, results in reduced currents running through NMDA receptor-activated ion channels; this causes neurons to fire less fast because there is less strong excitatory synaptic input, leading to shallower basins of attraction of the high firing-rate attractor states of the network^7^ (see Eq. 1 and Fig. 4).

The shallower basins of attraction arise firstly because with the neurons firing less fast, there is less positive feedback in the recurrent collateral connections between the neurons in the attractor, and this makes the system more vulnerable to noise (see Eq. 1). A second way in which reduced NMDA receptor function (or other factors such as synaptic pruning on the dendrites of cortical pyramidal cells^24,51^) could decrease the depth of the basins of attraction is by making the strengths of the synaptic connections (including a reduction in their number) between the neurons in the attractor weaker, which again reduces the positive feedback between the neurons in the attractor, and makes the attractor state more vulnerable to neuronal spiking-related noise. Decreases in excitatory synaptic efficacy and the number of spines that mediate excitatory transmission in the cortex using glutamate during late adolescence may be related to the onset of schizophrenia in those who are vulnerable^24,51^ and are prominent in the dorsolateral prefrontal cortex^52^ which is involved in short-term memory and attention^4,44,53,54^.

#### Negative symptoms

The negative symptoms represent a complex of symptoms including apathy, poor rapport, lack of spontaneity, motor retardation, disturbance of volition, blunted affect, and emotional withdrawal and passive behavior^37,39,40^. There are large individual differences in the magnitude of the negative symptoms in schizophrenia^55^. The negative symptoms and cognitive deficits are highly correlated in patients with schizophrenia and their non-psychotic relatives^56–58^. Moreover, Rolls and colleagues have found in a large-scale study with 2567 participants that the negative symptoms, as well as the positive and general symptoms, are reduced by treatment with antipsychotic drugs^55^. This is interesting as it may not occur with typical antipsychotic treatments (which have affinity particularly with D2 receptors), and may relate especially to the atypical antipsychotics that in addition modulate serotonin (5-HT), norepinephrine, and/or histamine neurotransmission^59^. Rolls and colleagues propose that the negative symptoms are also related to the decreased firing rates caused by a reduction in currents through NMDAR-activated channels, but in brain regions that may include the orbitofrontal cortex and anterior cingulate cortex^2,4,7,10,60,61^ rather than the dorsolateral prefrontal cortex. Indeed, lesions in these orbitofrontal and anterior cingulate brain areas are well known to produce symptoms that resemble the negative symptoms in schizophrenia, and neuronal firing rates and BOLD activations in these regions are correlated with reward value and pleasure^4,61,62^.

This is a unifying approach to the cognitive and negative symptoms: the same reduction in NMDAR-activated channel currents produces, on the one hand, instability in high-firing-rate states in attractor networks in the dorsolateral prefrontal cortex and thereby the cognitive symptoms, and on the other hand, a reduction in the firing rate of neurons in the orbitofrontal and cingulate cortex, leading to the negative symptoms. In addition to the reduced emotion caused by the reduced firing rates, attractor networks may be present in the orbitofrontal cortex that help to maintain mood state^2,62^, and a decrease in their stability by the reduced depth in the basins of attraction could make emotions more labile in schizophrenia/schizoaffective disorder.

#### Positive symptoms

The positive symptoms of schizophrenia include bizarre trains of thoughts, hallucinations, and delusions^37,39,55^. In contrast to the cognitive and negative symptoms, the positive symptoms generally occur intermittently during the course of the illness, and this clinical state is called “psychosis”. Rolls, Loh and Deco propose that owing to reduced currents through NMDAR-activated channels, the basins of attraction of the high-firing-rate attractor states are shallow^7,60,63^ in the temporal lobe, which includes the semantic memory networks and the auditory association cortex^4^. Because of the resulting statistical fluctuations in the states of the attractor networks, internal representations of thoughts and perceptions move too freely around in the energy landscape, from thought to weakly associated thought, leading to bizarre thoughts and associations, and to hallucinations (see Fig. 4). Such thoughts might eventually be associated together in semantic memory in the anterior temporal lobe, leading to false beliefs and delusions^4^.

In addition, Loh et al.^7^ propose that a reduction in GABA interneuron efficacy in schizophrenic patients may also contribute to the generation of positive symptoms: lower GABA-interneuron efficacy reduces the depth of the basin of attraction of the spontaneous state, making it more likely that a high firing-rate attractor state will emerge out of the spontaneous firing of the neurons (Fig. 4). This is illustrated in Fig. 3. On the spontaneous condition trial, the firing, which should have remained low throughout the trial as no cue was provided to start up the short-term memory, increased during the trial because of the statistical fluctuations, that is the spiking-related randomness in the network. This type of instability is more likely if GABA receptor-activated ion channel currents become decreased, or by other factors that decrease cortical inhibition. This type of instability in which a network jumps because of noise into a high firing rate state that is not triggered by an external input to the network (see Fig. 3) contributes it is suggested to the positive symptoms of schizophrenia, including for example hallucinations, delusions, and feelings of lack of control or being controlled by others^7,9,10^. Empirical evidence supports this computational proposal: markers indicating decreased inhibition by the GABA system in schizophrenia are found in neocortical areas^31,35,64–66^ and in parts of the hippocampus^67–69^ where it can impair brain function^69^. On the basis of this model, we have proposed^7,9^ that treating schizophrenia patients with D2 antagonists could increase the GABA currents^70,71^ in the networks, which would alleviate the positive symptoms by reducing the spontaneous firing rates, which would deepen the spontaneous attractor state (see Fig. 4). This cortical effect of D2 antagonists^53^ leaves the persistent (high firing rate) attractors shallow because the high firing rates are reduced, which may explain why the D2 antagonists do not have a major effect on the negative and cognitive symptoms. (The traditional view has been that the D2 antagonism of antipsychotic drugs is effective in the treatment of schizophrenia by acting in the striatum^33^.) The evidence for increased activity in the hippocampal system in schizophrenia might be related to reduced NMDA-receptor-based excitation of hippocampal GABA neurons^72^, which might increase the activity of hippocampal pyramidal cells. To target negative symptoms, we have suggested that D1 agonists (or other agents that facilitate glutamate transmission) may help to deepen the basin of attraction of the high-firing-rate attractor state^7,9,10^, and this action may also be relevant to the treatment of cognitive/working memory impairments in schizophrenia^36^. This two-dimensional (NMDA and GABA) approach allows us to address the specific characteristics of the psychotic (positive) symptoms which appear in episodes, in contrast to the negative and cognitive symptoms which typically persist over time.

When both NMDA and GABA are reduced one might think that these two counterbalancing effects (excitatory and inhibitory) would cancel each other out. However, this is not the case: modeling these conditions showed that the stability of both the spontaneous and the high-firing-rate states is reduced (Fig. 3)^7^ (see also^27,73^). Indeed, under these conditions, the network wandered freely between the two short-term memory (high firing rate) states in the network and the spontaneous state (Fig. 5). We relate this pattern to the positive symptoms of schizophrenia, in which both the basins of attraction of the spontaneous and high-firing-rate states are shallow, and the system jumps, helped by the statistical fluctuations, between the different attractor states and the spontaneous state (Figs. 3–5)^4,7,9^.

The evidence on GABA-mediated inhibition impairments in schizophrenia, and also of decreased spine density that would reduce excitatory transmission^31,50,66,74^, is an indication that the stability of cortical attractor networks is likely to be impaired in schizophrenia. The models described here have shown some of the effects that would be produced by altered levels of excitatory and inhibitory transmission on the stability of cortical circuitry, and how this might influence processes such as working memory and attention and produce some of the symptoms of schizophrenia.

### Reduced functional connectivity of some brain regions in schizophrenia

One way to investigate further the hypothesis that some networks in the brain are less stable in schizophrenia is to measure whether the functional connectivity between some brain regions is lower in schizophrenia. Functional connectivity can be measured by the Pearson correlation between the BOLD signal for each pair of brain regions over a time period of several minutes. A higher correlation is interpreted as showing that the nodes (the brain regions) are more strongly connected, in that they are influencing each other’s BOLD signals, or have a common input.

**Fig. 6:**
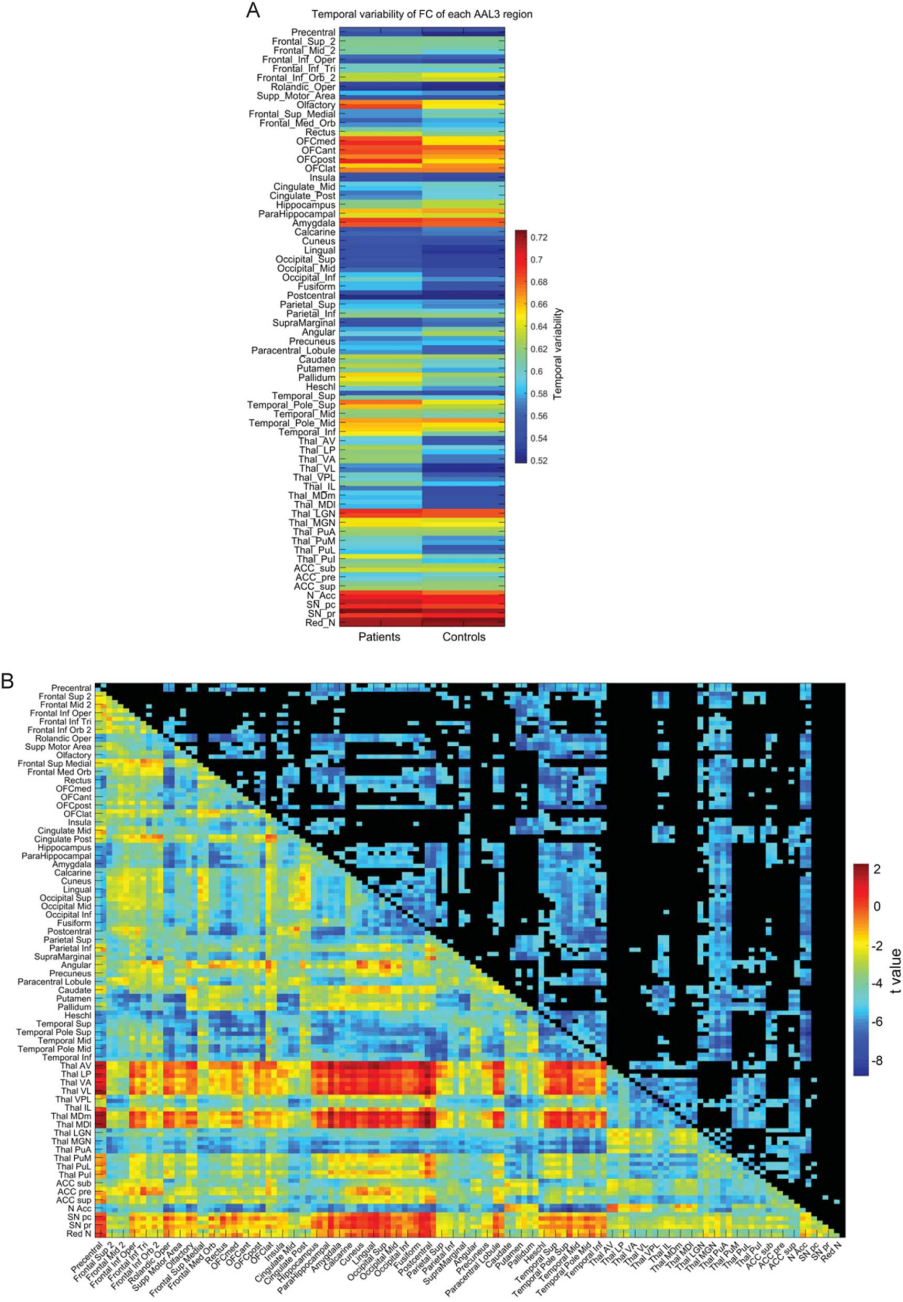
Increased temporal variability of functional connectivity in schizophrenia. **A** The temporal variability of the functional connectivity of different Automated Anatomical Labeling atlas 3 (AAL3) brain regions in chronic schizophrenic and control groups. The temporal variability of the functional connectivity measures how much the functional connectivity of a brain region with other brain regions changes across time^75,78^. **B** The mean (across time) functional connectivity of AAL3 areas for the chronic schizophrenic group minus controls. The lower left shows the *t* value for the difference in functional connectivity of patients—controls; the upper right shows the significance after Bonferroni correction. The functional connectivity is measured by the Pearson correlation between the BOLD signals in a pair of brain regions. The brain regions shown are from the AAL3 atlas^76^. (After Rolls et al.^75^).

From one such investigation, the resting-state functional connectivity in a group of 123 patients with chronic schizophrenia compared to 136 matched healthy controls is shown in Fig. 6B^75^. The matrix shows the functional connectivity differences for pairs of brain areas from the anatomical labeling atlas 3^76^. First, it is evident that many of the functional connectivities are significantly lower in schizophrenia. This is consistent with the hypothesis that the level of excitation between cortical areas is lower in schizophrenia, which is equivalent in the simulations described above to a reduction in the NMDA synaptic conductances. This is consistent with the disconnectivity hypothesis of schizophrenia^77^.

Moving beyond the disconnectivity hypothesis, the reduced functional connectivities evident in Fig. 6B might lead us to expect that there might be signs of less stability in the BOLD signal in schizophrenia. This was shown to be the case, in that the temporal variability of the functional connectivities of many of the brain regions was higher in schizophrenia, as shown in Fig. 6A. (The temporal variability of functional connectivity measures how much the functional connectivity of a brain region with other brain regions changes across time^75^.) The higher temporal variability was especially clear for some early visual cortical areas (Inferior Occipital and Fusiform), the temporal lobe areas connected to these, and the orbitofrontal cortex. This is an indication of increased instability of these brain regions in schizophrenia^75^.

Very interestingly, this higher temporal variability of functional connectivity reflecting the instability of some early visual cortical areas, the temporal lobe areas connected to these, and the orbitofrontal cortex, which could be related to lower functional connectivities of especially these areas, as shown in Fig. 6B. Especially interesting was that the functional connectivities of the sensory thalamic visual relay, the lateral geniculate nucleus, and the thalamic sensory auditory relay, the medial geniculate nucleus, were lower in schizophrenia (Fig. 6B). This was in interesting contrast to the association thalamic nuclei, which had increased functional connectivity in schizophrenia^75^. This finding was cross-validated in a different set of patients with first-episode schizophrenia, who had similar though somewhat smaller differences from controls^75^.

These findings are consistent with the hypothesis that a factor in schizophrenia is a reduction in the connectivity and therefore excitability of some brain regions, which destabilizes attractor networks in these regions because the firing rates are insufficient to maintain the networks in a high firing rate state. This results in increased temporal variability of the functional connectivity between brain regions, which can be directly related to the symptoms of reduced maintenance of attention, and increased mind-wandering and thought associations in schizophrenia^75,78^. In addition, we propose that in schizophrenia these differences bias processing away from external visual and auditory inputs, and towards internal cognitive processing in associative cortical areas such as the prefrontal and temporal cortical areas. We relate this to the tendency for people with schizophrenia to be disconnected from the world and to be unable to maintain attention^75^. This relates the phenomenology of schizophrenia to the underlying differences of connectivity and the associated brain dynamics^75^. There is evidence that the temporal dynamics of the brain operate close to a critical point as shown by the scale-free synchronized peaks or avalanches in the BOLD signal from many different brain areas, which are reciprocally related to the temporal variability of the functional connectivity^78^. The hypothesis is that in schizophrenia the brain is operating with increased temporal variability and correspondingly reduced synchronized peaks of activity, and is thus at a different operating point of the dynamics^75,78^.

### Beyond the disconnectivity hypothesis of schizophrenia: reduced forward but not backward connectivity

It has been possible to go beyond the disconnectivity hypothesis of schizophrenia^77^, not only in terms of reduced dynamical stability of early visual cortical and related areas as described above^75^ but also in terms of the direction of the connectivities that are decreased, as described next^79^.

In hierarchical cortical systems, the forward connectivities up through the hierarchy are strong, to drive the processing up through the hierarchy; and the back projections are weaker, as they are used for memory recall and for top-down attentional bias^3^. Measurements can be made of the connectivity in each of these directions, by making use of differences in the signals between successive timesteps. The connectivity in each direction is termed *effective connectivity*. To investigate how the directed or effective connectivities are different in schizophrenia, to see whether they are different for particular brain areas, or in particular directions, we have analyzed effective connectivity in schizophrenia, comparing the resting state effective connectivity in 181 participants with schizophrenia and 208 controls^79^.

The first key finding was that for the significantly different effective connectivities in schizophrenia, on average the forward (stronger) effective connectivities were smaller, but the backward connectivities tended to be larger, in schizophrenia, and the difference was significant^79^. An implication of this is that the feedforward sensory inputs from the world are less effective in schizophrenia; and that the top-down backward connectivities that mediate the effects of memory recall and attention^3^ show little difference in schizophrenia. This would tend to disconnect the individual from the world, and enclose the individual in an imaginary world too dominated by internal representations not corrected towards reality by sensory information from the world. Put in another way, if top-down signals are increased relative to bottom-up signals this would increase the importance of priors, i.e., beliefs, at the cost of sensory signals, representing a possible mechanism for the emergence of hallucinations and delusions^80^.

A second key finding in schizophrenia was the high effective connectivity directed away from the precuneus and the closely related posterior cingulate cortex^79^. The connectivity in the strong (or forward) direction in schizophrenia to the precuneus is similar to that in the healthy controls, and it is in the weak (back projection) direction that the effective connectivity is higher in schizophrenia than in controls. It is suggested that by influencing other areas too much by its back projections, the precuneus may contribute to the symptoms of schizophrenia. The areas to which the back projections from the precuneus and posterior cingulate cortex are higher in schizophrenia than in controls include the parahippocampal and hippocampal cortices^79^.

I, therefore, consider how these differences in the connectivity of the precuneus and posterior cingulate cortex are involved in schizophrenia. The precuneus is a medial parietal cortex region implicated in the sense of self, agency, autobiographical memory, and spatial function^81,82^, and this may relate to the altered sense of self that is a feature of schizophrenia. The precuneus and the adjoining retrosplenial cortex (areas 29 and 30)^83^ are key regions related to spatial function, memory, and navigation^4,81,82,84–87^. The retrosplenial cortex provides connections to and receives connections from the hippocampal system, connecting especially with the parahippocampal gyrus areas TF and TH, and with the subiculum^84,88^. The precuneus can be conceptualized as providing access to the hippocampus for spatial and related information from the parietal cortex (given the rich connections between the precuneus and parietal cortex and even the hippocampus^88^). This increased effective connectivity from the precuneus to the hippocampal system is of special interest as it may contribute to the overactivity of the hippocampus in schizophrenia, which is consistent with the high Sigma parameter reflecting signal variance in schizophrenia also found for the hippocampus^79^. Further, the precuneus has rich connectivity with the posterior cingulate cortex, which provides a pathway into the hippocampal memory system^4,87,88^. The precuneus is part of the default mode network, which becomes more active when tasks are not being performed in the world, and instead, internal thoughts and processing are occurring.

The posterior cingulate cortex is also a key region of the default mode network with strong connectivity in primates with the entorhinal cortex and parahippocampal gyrus, and thus with the hippocampal memory system^4,61^. The posterior cingulate region (including the retrosplenial cortex) is consistently engaged by a range of tasks that examine episodic memory including autobiographical memory, and imagining the future, and also spatial navigation and scene processing^61,89,90^.

The proposal made based on the findings described here and the evidence about the functions of the precuneus and posterior cingulate cortex is that the high back-projection effective connectivities from the precuneus may relate to increased internal thoughts about the self in schizophrenia, the world in which the self exists, and the relatively greater role of these internal thoughts which are not dominated by the sensory inputs from the word which normally keep the self in contact with the real world and with real-world inputs. Correspondingly, it was proposed that the high back-projection effective connectivities from the posterior cingulate cortex in schizophrenia may relate to increased memory-related internal thoughts involving relatively higher dominance of memories over the normal forward real-world sensory inputs that normally keep us in contact with the real world^79^. These over-connected brain systems could contribute to the delusions and thought disorders that are part of the positive symptoms of schizophrenia.

Thus overall we have seen how concepts about the stability and connectivity of cortical networks can be applied to help understand some important aspects of a key mental disorder, schizophrenia^4^.

## Depression and attractor dynamics

### Depression, non-reward, and the orbitofrontal cortex

Major depressive episodes, found in both major depressive disorder and bipolar disorder, are pathological mood states characterized by persistently sad or depressed mood^12,91^. Major depressive disorders are generally accompanied by (1) altered incentive and reward processing, evidenced by motivation, apathy, and anhedonia; (2) impaired modulation of anxiety and worry, manifested by generalized, social, and panic anxiety, and oversensitivity to negative feedback; (3) inflexibility of thought and behavior in association with changing reinforcement contingencies, apparent as ruminative thoughts of self-reproach, pessimism, and guilt, and inertia toward initiating goal-directed behavior; (4) altered integration of sensory and social information, as evidenced by mood-congruent processing biases; (5) impaired attention and memory, shown as performance deficits on tests of attention set-shifting and maintenance, and autobiographical and short-term memory; and (6) visceral disturbances, including altered weight, appetite, sleep, and endocrine and autonomic function. This section describes an attractor-based theory of some of the brain mechanisms that are related to depression^13^, and tests of the theory^11^.

**Fig. 7:**
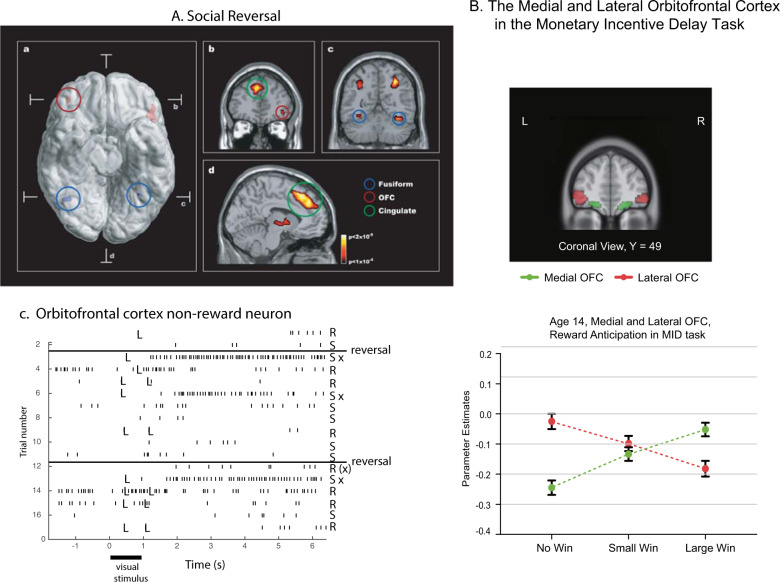
Non-reward in the orbitofrontal cortex. **A** The human lateral orbitofrontal cortex is activated by non-reward in a visual discrimination reversal task on reversal trials, when a face was selected but the expected reward was not obtained, indicating that the participant should select the other face in the future to obtain the reward. **a** A ventral view of the human brain with an indication of the location of the two coronal slices (**b**, **c**) and the transverse slice (**d**). The activations with the red circle in the lateral orbitofrontal cortex (OFC, peaks at [42 42–8] and [−46 30–8]) show the activation on reversal trials compared to the non-reversal trials. For comparison, the activations with the blue circle show the fusiform face area produced just by facial expressions, not by reversal, which is also indicated in the coronal slice in (**c**). **b** A coronal slice showing the activation in the right orbitofrontal cortex on reversal trials. Activation is also shown in the supracallosal anterior cingulate region (Cingulate, green circle) that is also known to be activated by many punishing, unpleasant, stimuli (see Grabenhorst and Rolls^97^) (From Kringelbach et al.^93^). **B** Activations in the human medial orbitofrontal cortex (OFC) are related to Wins, and in the lateral orbitofrontal cortex to non-reward (No Win) in the monetary incentive delay task. The data are from 1877 participants aged 14 years, with similar results at age 19. (Modified from Xie et al.^96^). **C** Non-reward error-related neurons maintain their firing after non-reward is obtained. Responses of an orbitofrontal cortex neuron that responded only when the macaque licked to a visual stimulus during reversal, expecting to obtain fruit juice reward, but actually obtained the taste of aversive saline because it was the first trial of reversal (trials 3, 6, and 13). Each vertical line represents an action potential; each L indicates a lick response in the Go-NoGo visual discrimination task. The visual stimulus was shown at time 0 for 1 s. The neuron did not respond on most reward (R) or saline (S) trials but did respond on the trials marked S x, which were the first or second trials after a reversal of the visual discrimination on which the monkey licked to obtain the reward, but actually obtained saline because the task had been reversed. The two times at which the reward contingencies were reversed are indicated. After responding to non-reward, when the expected reward was not obtained, the neuron fired for many seconds and was sometimes still firing at the start of the next trial. It is notable that after an expected reward was not obtained due to a reversal contingency being applied, on the very next trial the macaque selected the previously non-rewarded stimulus. This shows that rapid reversal can be performed by a non-associative process, and must be rule-based. (After Thorpe et al.^92^).

The attractor theory of depression starts with the evidence that the orbitofrontal cortex contains a population of error neurons that respond when an expected reward is not obtained and maintain their firing for many seconds after the non-reward, providing evidence that they have entered an attractor state that maintains a memory of the non-reward^4,12,92^. An example of such a neuron is shown in Fig. 7C. The human lateral orbitofrontal cortex is activated by non-reward during reward reversal^93,94^ (Fig. 7A), by losing money^95^ or not winning^96^ (Fig. 7B), and by many other aversive stimuli^97^. Further evidence that the orbitofrontal cortex is involved in changing rewarded behavior when non-reward is detected is that damage to the human orbitofrontal cortex impairs reward reversal learning, in that the previously rewarded stimulus is still chosen during reversal even when no reward is being obtained^98–100^. Further, the right lateral orbitofrontal cortex is strongly activated by non-reward in a one-trial rule-based reward reversal task^94^, which is the same brain region with increased functional connectivity in depression as described below.

Now it is well established that not receiving an expected reward, or receiving unpleasant stimuli or events, can produce feelings of depression^12,101–104^. A clear example is that if a member of the family dies, then this is the removal of reward (in that we would work to try to avoid this), and the result of the removal of the reward can be depression. More formally, in terms of learning theory, the omission or termination of a reward can give rise to sadness or depression, depending on the magnitude of the reward that is lost, if there is no action that can be taken to restore the reward^2,12,105^.

### A non-reward attractor theory of depression

The theory has been proposed that in depression, the lateral orbitofrontal cortex non-reward/punishment attractor network system is more easily triggered, and maintains its attractor-related firing for longer^4,12,13,106,107^. The greater attractor-related firing of the non-reward/punishment system triggers negative cognitive states held online in other cortical systems such as the language system and in the dorsolateral prefrontal cortex which is implicated in attentional control. These other cortical systems then in turn have top-down effects on the orbitofrontal cortex non-reward system that bias it in a negative direction^108^, and thus increase the sensitivity of the lateral orbitofrontal cortex to non-reward and maintain its overactivity^13^. It is proposed that the interaction of non-reward and language/attentional brain systems of these types accounts for the ruminating and continuing depressive thoughts, which occur as a result of a positive feedback cycle between these types of brain system^13^. It is argued that paying attention to depressive symptoms when depressed may in this way exacerbate the problems in a positive feedback way^13^.

More generally, the presence of the cognitive ability to think ahead and see the implications of recent events that are afforded by language may be a computational development in the brain that exacerbates the vulnerability of the human brain to depression^12,13^. For example, with language, we can think ahead and see that perhaps the loss of an individual in one’s life may be long-term, and this thought and its consequences for our future can become fully evident^4^.

The theory is that one way in which depression could result from over-activity in this lateral orbitofrontal cortex system is if there is a major negatively reinforcing life event that produces reactive depression and activates this system, which then becomes self-re-exciting based on the cycle between the lateral orbitofrontal cortex non-reward/punishment attractor system and the cognitive/language system, which together operate as a systems-level attractor^13^. (The generic cortical architecture for such reciprocal feedforward and feedback long loop excitatory attractor effects is illustrated by Rolls^3^.)

The theory is that a second way in which depression might arise is if this lateral orbitofrontal cortex non-reward/punishment system is especially sensitive in some individuals. This might be related for example to genetic predisposition, or to the effects of stress^12,109^. In this case, the orbitofrontal cortex system would over-react to normal levels of non-reward or punishment, and start the local attractor circuit in the lateral orbitofrontal cortex, which in turn would activate the cognitive system, which would feedback to the over-reactive lateral orbitofrontal cortex system to maintain now a systems-level attractor with ruminating thoughts^13^. This is described as a “systems-level” attractor because it includes mutual excitations between different brain areas^3^.

Given that the activations of the lateral and medial orbitofrontal cortex often appear to be reciprocally related^95,96,110^ (Fig. 7B), the other part of the theory of depression is that in depression there may be underactivity, under-sensitivity, or under-connectivity of the (reward-related) medial orbitofrontal cortex in depression^12,13^. The theory is further that under-responsiveness of the medial orbitofrontal cortex could contribute to other aspects of depression, such as anhedonia.

### The orbitofrontal cortex, and the theory of depression

This approach to understanding depression has been investigated by large-scale neuroimaging studies of functional connectivity and brain activations in people with depression vs controls^11^.

**Fig. 8:**
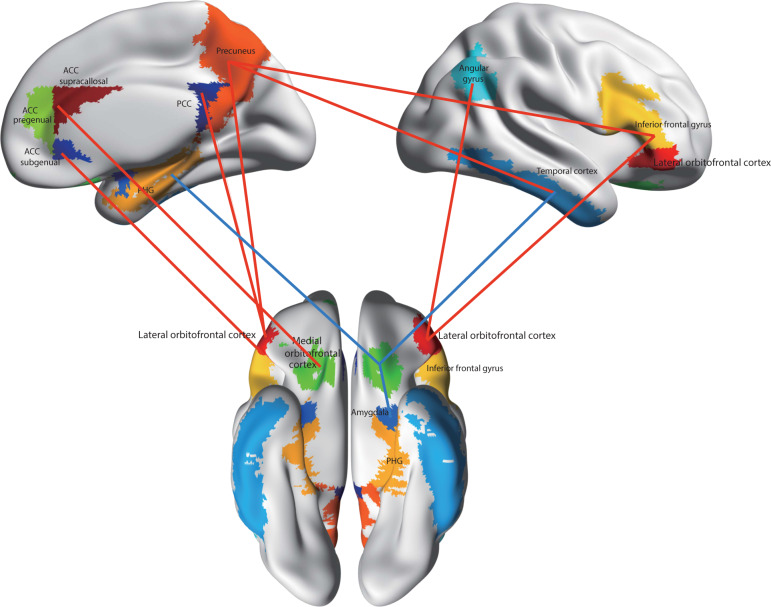
Functional connectivity (FC) differences of the medial and lateral orbitofrontal cortex in major depressive disorder. Higher functional connectivity in depression is shown by red connecting lines and includes higher functional connectivity of the non-reward/punishment-related lateral orbitofrontal cortex with the precuneus, posterior cingulate cortex (PCC), pregenual anterior cingulate cortex (ACC), angular gyrus, and inferior frontal gyrus. Lower functional connectivity in depression is shown by blue connecting lines and includes lower functional connectivity of the medial orbitofrontal cortex with the parahippocampal gyrus memory system (PHG), amygdala, temporal cortex, and supracallosal anterior cingulate cortex (ACC). The part of the medial orbitofrontal cortex in which voxels were found with lower functional connectivity in depression is indicated in green. The areas apart from the medial orbitofrontal cortex shown are as defined in the automated anatomical labeling atlas^124^, although the investigations that form the basis for the summary were at the voxel level. (After Rolls et al.^11^).

In the first brain-wide voxel-level resting-state functional-connectivity neuroimaging analysis of depression (with 421 patients with major depressive disorder and 488 controls), we have found that one major circuit with altered functional connectivity involved the medial orbitofrontal cortex BA 13, which had reduced functional connectivity in depression with memory systems in the parahippocampal gyrus and medial temporal lobe^111^ (Fig. 8). The lateral orbitofrontal cortex BA 12/47, involved in non-reward and punishing events, did not have this reduced functional connectivity with memory systems so that there is an imbalance in depression towards decreased reward-related memory system functionality.

A second major circuit change was that the lateral orbitofrontal cortex area BA 12/47 had increased functional connectivity with the precuneus, the angular gyrus, and the temporal visual cortex BA 21^111^ (Fig. 8). This enhanced functional connectivity of the non-reward/punishment system (BA 12/47) with the precuneus (involved in the sense of self and agency), and the angular gyrus (involved in language) is thus related to the explicit affectively negative sense of the self, and of self-esteem, in depression.

The differences in orbitofrontal cortex connectivity with these brain regions were related to the depression by evidence that the symptoms of depression were correlated with these differences of functional connectivity; and that the lateral orbitofrontal cortex functional connectivity links described were less high if the patients were receiving antidepressant medication^111^.

The reduced functional connectivity of the medial orbitofrontal cortex, implicated in reward, with memory systems provides a new way of understanding how memory systems may be biased away from pleasant events in depression. The increased functional connectivity of the lateral orbitofrontal cortex, implicated in non-reward and punishment, with areas of the brain implicated in representing the self, language, and inputs from face and related perceptual systems provides a new way of understanding how unpleasant events and thoughts, and lowered self-esteem, may be exacerbated in depression^111,112^.

These differences of functional connectivity are related to the orbitofrontal cortex attractor theory of depression^13,106^ because increased functional connectivity of the non-reward lateral orbitofrontal cortex would increase the stability and persistence of its negative attractor mood-related states; and decreased functional connectivity of the reward-related medial orbitofrontal cortex would decrease the stability and persistence of its positive mood states^11,12^.

These advances have stimulated many other large-scale voxel-level investigations of functional connectivity in depression, which develop these hypotheses further^4,11,112–118^ and provide cross-validation^119^.

### Activations of the orbitofrontal cortex related to depression

It is also of interest to examine whether the sensitivity of the orbitofrontal cortex to reward and non-reward is different in depression, as another test of the theory of depression^13^.

In 1140 adolescents at age 19 and 1877 at age 14 in the monetary incentive delay task, we found that the medial orbitofrontal cortex had graded increases in activation as the reward (Win) value increased^96^. The lateral orbitofrontal cortex had graded increases of activation as the reward value dropped to zero (the No-Win condition) (Fig. 7B).

In a subgroup with a high score on the Adolescent Depression Rating Scale at age 19 and 14, the medial orbitofrontal cortex activations had reduced sensitivity to the different reward conditions; and the lateral orbitofrontal cortex activation showed high activation to the No-Win (i.e., Non-reward) condition^96^. These new findings provide support for the hypothesis that those with symptoms of depression have increased sensitivity to non-reward in the lateral orbitofrontal cortex, and decreased sensitivity for differences in reward of the medial orbitofrontal cortex. Moreover, these differences are evident at an age as early as 14 years old^96^. This increase in Non-reward sensitivity of the lateral orbitofrontal cortex in depression, and decreased Reward sensitivity of the medial orbitofrontal cortex, may act together with the altered functional connectivity of these regions just described, to make some individuals susceptible to depression^11^.

It is hypothesized that as part of the process of evolution, variation of the sensitivity of individuals to specific types of Reward and Non-Reward may be present^2^. Individuals with high sensitivity to Non-Reward may be susceptible to depression, and individuals with low sensitivity to Non-Reward may be impulsive because they are little affected by non-reward^2,12^. Individuals with high sensitivity to Reward may be sensation-seekers (with increased functional connectivity of the medial orbitofrontal cortex, and for that reason also impulsive^120^), and individuals with low sensitivity to Reward may have reduced goal-seeking behavior and reduced motivation^2,12^. These types of natural variation may be important foundations for different types of personality^2,121^ and may relate to why some individuals are more susceptible to depression.

### Implications for the treatment of depression

One implication of the approaches described here is that the orbitofrontal cortex may be a key brain area to focus on when developing treatments for depression, whether as a marker for the effects of different types of treatment, or possibly for intervention studies^11,12^. The orbitofrontal cortex is a key brain region in emotion and provides a foundation it is suggested for understanding some disorders of emotion, including depression^2,11,12^. Another implication is that whereas current antidepressant medications reduce the elevated functional connectivity of the non-reward-related lateral orbitofrontal cortex, they do not ameliorate the reduced functional connectivity of the reward-related medial orbitofrontal cortex^11,12,113^. That suggests that there is scope for the development of new treatments that normalize the operation of the medial orbitofrontal cortex, and perhaps treat especially symptoms such as the anhedonia of depression. Another implication is that the orbitofrontal cortex may be a key brain area for electrical brain stimulation reward that may alleviate depressed mood^11^. Another implication is that especially on the right, the lateral orbitofrontal cortex non-reward system implicated in depression extends around the inferior convexity to the right inferior frontal gyrus that is part of the lateral orbitofrontal cortex area 12^11,94,113,114^, and this extended lateral orbitofrontal cortex region should be considered. Another implication is that by better understanding depression in relation to differences in reward and non-reward systems in the brain related to emotion, and how these relate to the rational (reasoning) systems in our brains^2,122,123^, purely cognitive ways of ameliorating depression and reducing sad rumination can be encouraged^12^.

## Conclusions

This contribution shows how understanding differences in the stability of attractor network systems in different brain areas can help to provide a scientific basis for relating some mental disorders to the operation of the underlying brain systems^4^. These advances in turn have implications for treatments. Complementary analyses have related increased glutamate-mediated excitatory function to attractor over-stability of attractor networks in obsessive–compulsive disorder^10,29^.
